# Supplementary material for: Causal interplay between lactose intolerance and gut microbiota: a combined bidirectional Mendelian randomization and in vivo validation study
Source: Front Nutr. 2026 Jun 1;13:1803337. doi: 10.3389/fnut.2026.1803337 (PMC13265576; doi:10.3389/fnut.2026.1803337)
Supplement: Supplementary file 1 [file Data_Sheet_1.zip › supplementary materials/Forward/leave one out cross validation/ebi-a-GCST90027568.finngen_R12_E4_LACTONAS.pdf]

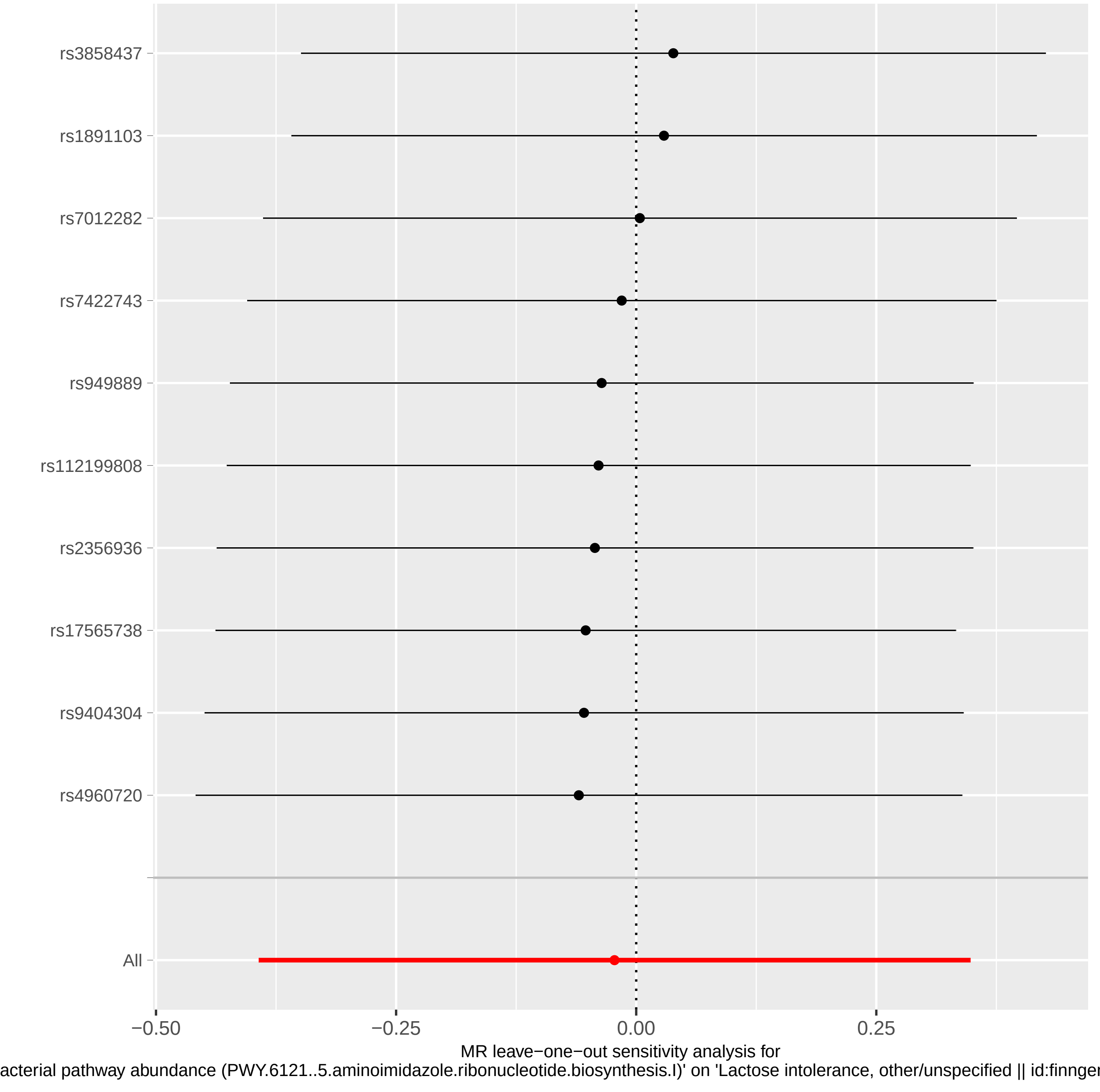

rs3858437
rs1891103
rs7012282
rs7422743
rs949889
rs112199808
rs2356936
rs17565738
rs9404304
rs4960720
All
−0.50
−0.25
0.00
0.25
MR leave−one−out sensitivity analysis for
acterial pathway abundance (PWY.6121..5.aminoimidazole.ribonucleotide.biosynthesis.I)' on 'Lactose intolerance, other/unspecified || id:finnger
